# Programmes for the prevention of mother-to-child HIV infection transmission have made progress in Yunnan Province, China, from 2006 to 2015: a cost effective and cost-benefit evaluation

**DOI:** 10.1186/s12879-019-3708-x

**Published:** 2019-01-17

**Authors:** Xiaowen Wang, Guangping Guo, Jiarui Zheng, Lin Lu

**Affiliations:** 1 Yunnan Center for Disease Control and Prevention, No.158, Dongsi Street, Xishan District, Kunming, 650022 Yunnan Province China; 2 Department of Public Health, Kunming Medical University, No. 1168, West Chunrong Road, Yuhua Street, Chenggong District, Kunming, 650599 Yunnan Province China; 3 Yunnan Maternal and Child Health Care hospital, No. 200, Gulou Road, Wuhua District, Kunming, 650032 Yunnan Province China; 4 Health and Family Planning Commission of Yunnan Province, No. 309, Guomao Street, Kunming, 650299 Yunnan Province China

**Keywords:** Prevention mother-to-child transmission of HIV, Economic evaluation, Cost effective, Cost benefit, Yunnan Province

## Abstract

**Background:**

Prevention of mother-to-child transmission (PMTCT) of HIV programmes have substantially reduced HIV infections among infants in Yunnan Province, China. We conducted a macro-level economic evaluation of Yunnan’s PMTCT programmes over the 10 years from 2006 to 2015 from a policymaker perspective.

**Methods:**

The study methodology was in accordance with the guidelines from the Consolidated Health Economic Evaluation Reporting Standards (CHEERS) statement. We quantified the output from the Yunnan’s PMTCT programmes by estimating the number of paediatric HIV infections averted and the relative savings to both the health care system and society. The return-on-investment ratio (ROI) was calculated as the output (numerator) divided by the input (denominator).

**Results:**

We have found that the US$ 49 million investment in Yunnan’s PMTCT programmes over the period from 2006 to 2015 averted an estimated 2725 new paediatric HIV infections and resulted in an estimated 134,008 QALY acquired. It saved an estimated US$ 0.5 billion in treatment expenditures for Yunnan’s healthcare system and nearly US$ 3.9 billion in productivity. The ROI was 88.4, meaning every US$ 1 invested brought about US$ 88.4 in benefits.

**Conclusions:**

Our results support the ongoing investment in PMTCT programmes in Yunnan Province. The PMTCT strategy is a cost effective and cost-benefit strategy in the periods from 2006 to 2015. Despite higher investments in the future, the overall investment in the PMTCT programmes in Yunnan province could be offset by averting more paediatric infections.

## Background

Prevention of mother-to-child transmission (PMTCT) of HIV infection became a key public health priority in China in 2002 [1]. In the fifteen years since 2002, China’s HIV PMTCT programmes have played an important role in the prevention of HIV infection nationwide. A meta-analysis has shown that the mother-to-child transmission (MTCT) rate in China declined from 12.90 to 2.29% from 2004 to 2010, demonstrating a decrease year-by-year under the PMTCT strategies [2]. In 2017, China began working towards an official certification for the elimination of HIV transmission from mother-to-child via the World Health Organization.

In southwest China’s Yunnan province, the number of people living with HIV and AIDS was estimated to be 93,437 at the end of 2016 [3] (total population of 4.7 million in Yunnan Province in 2016 [4]), the highest among all provinces and municipalities. PMTCT programmes have been carried out since 2003 in Yunnan Province. With more than one decade of development and efforts, these programmes have been shown to be successful. Previous studies conducted by various cities and counties in Yunnan province have shown that the coverage rate of PMTCT programmes is nearly 100% in their own areas [5–14]. Based on report data from Yunnan Provincial Maternal and Infant Health Care Centre, the MTCT rate declined from 34.80% in 2003 to 3.13% in 2015 throughout the province.

However, although the clinical and public health benefits of Yunnan’s PMTCT programmes are relatively well understood, the relationship between these benefits and their economic costs and savings has not been comprehensively studied. It is known that the economic burden of HIV infection is substantial [15]. A clear and objective assessment of the return on investment due to PMTCT programmes is an important input in the decision-making process for health planners and policymakers. Tasked with allocating limited resources, health planners and policymakers must make informed evaluations whether the further scale-up of PMTCT programmes will be a cost-effective means for averting paediatric HIV infections and whether it will make good use of prevention resources [16–19]. Therefore, we primarily aimed to evaluate PMTCT programmes province-wide at the macro level by retrospectively quantifying the return on investment about Yunnan province’s PMTCT programmes over the 10 years from 2006 to 2015. Second, we aimed to estimate the historical economic impacts of the programmes from a policymaker perspective.

## Methods

The methodology we applied in the study was in accordance with the Consolidated Health Economic Evaluation Reporting Standards (CHEERS) statement guidelines [20].

### Study overview

PMTCT services were provided by different maternal and infant healthcare centres at their respective administrative levels in Yunnan province. The target population of PMTCT programmes was pregnant women living with HIV (including those with infants less than 18 months old). PMTCT services were first piloted in Yunnan province in 2003. According to the regimens commented by the WHO, single-dose nevirapine was provided to HIV-infected pregnant women. In 2005, PMTCT programmes began to scale up and expand. The regimens were changed to zidovudine with nevirapine for HIV-infected pregnant women starting from 14 weeks of gestation until delivery. Newborns were given 42 days of nevirapine with formula feeding, which helped to reduce the risk of HIV transmission from 34.80 to 11.36%, approximately reduced by 2/3. The PMTCT regimen changed again to a triple-drug regimen in 2008, which contained zidovudine, efavirenz and lopinavir/ritonavir. Since 2015, WHO Option B+ has been included in Yunnan PMTCT programmes, which will hopefully encourage Yunnan province to move towards achieving the elimination certification. Table 1 describes the services and interventions offered during the Yunnan PMTCT programmes’ major development period.

**Table 1:** Main activities/interventions of each stage’s PMTCT programs in Yunnan Province, China

Year	Activities/interventions
2003	1. Voluntary counseling and testing (VCT). 2. Safe delivery services. 3. Feeding counseling services. 4. HIV-antibody testing for infants with the age of 12 and 18 months old.
2004–2007	1. Health education. 2. Counselting and testing services. 3. ARV or ART (CD4 + T cell< 250, starting ART, AZT + 3TC + NVP; CD4 + T cell≥250, AZT + NVP before, during and after pregnancy or NVP after pregnancy). 4. Safe delivery services (HIV positive not the only indication of the cesarean). 5. Encourage formula feeding, avoid breast feeding and forbid mixed feeding.
2008–2010	1. Counseling and testing services. 2. Health care for pregnant women with HIV. 3. ART (with no indication of ART or having not accepted ART before, before paregnancy: AZT; during pregnancy: AZT + NVP + 3TC; after: AZT). 4. Safe delivery services. 5. Provide formula feeding, avoid breast feeding and forbid mixed feeding. 6. Supply supporting and care services.
2011–2014	1. Provide AIDS, Syphilis and Hepatitis B counseling and testing services (Opt-out). 2. Select the pregnancy outcomes. 3. Supply PMTCT services to pregnant women with HIV and their children (including ART, (whatever CD4+ T cell counts, recommendation ART, AZT + 3TC + LPV/r or AZT + 3TC + EFV or CD4 + Tcell≤350, ART after pregnancy, CD4 + Tcell> 350, stopping ART,) safe delivery services, scientific feeding guiding and counseling (advocate formula feeding, avoid breast feeding and forbid mixed feeding)). 4. Supply AIDS early diagnosis for infants born to pregnant women with HIV/AIDS and supply ARV to them to prevent opportunistic infection.
2015-Nowadays	1. Health education and health promotion. 2. Active counseling and testing services (Opt-out). 3. PMTCT services (including ATR (whatever CD4 + T cell counts, start ART at once after infomed consent), safe delivery services, ARV for infants, cientific feeding guiding and counseling (advocate formula feeding, avoid breast feeding and forbid mixed feeding)). 4. Supply AIDS early diagnosis and HIV-antibody testing for infants born to pregnant women with HIV/AIDS.

A cost-effective analysis was conducted from the perspective of the health system, and a cost-benefit analysis was conducted from the perspective of policymakers.

Information on the investment in Yunnan’s PMTCT programmes was obtained from the Yunnan AIDS Prevention Bureau. Annual investments from all sources over the time period from 2006 to 2015 were summed and used in further economic impact analyses. All investments in PMTCT strictly obeyed the regular use solely for PMTCT services. The basic costs incurred by this programme are fixed asset purchasing (including medical equipment and office equipment), ability building (including technical personnel training, supervisor and evaluation), interventions (including health education, inspector subsidizing, pregnant women screening, CD4+ testing, correct diagnosing of HIV-infected pregnant women, ART for HIV-infected pregnant women, early diagnosis of newborns, formula feeding, care and assistance for HIV-infected pregnant women in poor conditions and follow-up). All investments and costs from 2006 to 2015 were first estimated in 2016 Chinese Yuan (CN¥). They were then converted to 2016 United States Dollars (US$) using the 23 November 2016 exchange rate (CN¥6.8916 = US$1.0000). The average inflation rate in China in the last 5 years ranged from 0.0 to 5.0% [21]. Based on the commendation from the World Health Organization CHOosing Interventions that are Cost-Effective (WHO-CHOICE), a discount rate of 3.0% was used to convert the economic costs and health outcomes in the baseline analysis [22]. A discount rate of 0.0 and 5.0% was used in the sensitivity analysis. The mother-to-child transmission (MTCT) rates used in the calculations of paediatric HIV infections averted were estimated using HIV surveillance data from the Yunnan Maternal and Infant Health Care Centre. Because the study was based on aggregate data, ethical approval was not required. All data used in this study were obtained through approvals from Yunnan AIDS Prevention Bureau.

### Outcome measures

The outcomes of our study included paediatric HIV cases averted, quality of life years (QALY) acquired and economic costs avoided.

### Paediatric HIV cases averted

We used the difference between the HIV MTCT rates in the pre-PMTCT era (mean rate from 2003 to 2005 in Yunnan counties with no pilot PMTCT services) and the post-PMTCT era (mean rate from 2006 to 2015 across all of Yunnan province after PMTCT services had been scaled up) to estimate the total number of paediatric HIV cases averted. The total estimated number of paediatric cases averted was compared to investments made over the same 10-year time period to generate an estimated cost per averted infection. Because the PMTCT interventions that we compared in the study were all carried out in Yunnan Province itself, we assumed a pre-PMTCT era with no other related programmes or very minimal programmes implemented for the prevention of mother-to-child transmission of HIV as the base for the comparison.

### QALY acquired

The net number of QALY acquired by a single averted paediatric HIV infection was a function of the difference between the expected number of QALY of a child without HIV infection and the excepted number of QALY of a child with HIV infection [23]. We adopted the health utilities of 0.74 for the HIV sample [24] and of 1 for the general sample to weight the life years gained and the determined QALY acquired. Based on a previous study [25], we assumed the life expectancy of a treated HIV-infected child to be 28.79 years. Normal life expectancy for children born in Yunnan Province is 70.5 years [26]. The total estimated QALY acquired was compared to investments made over the same 10-year time period to generate an estimated cost per QALY gained.

### Economic costs avoided

We calculated the total economic cost of a single paediatric HIV case as the sum of direct and indirect costs. Direct costs were defined as life-long expenses associated with HIV/AIDS treatment (mainly comprising the costs of regular testing and follow-up, ART, and the prevention, diagnosis, and treatment of opportunistic infections [27]). Direct costs were first estimated for adults and then adjusted for children by applying a multiplier of 1.75 [28]. Indirect costs were equated to the value of productivity lost due to early HIV/AIDS-related death and estimated in four steps. First, the age range during which members of society contribute to the labour force was assumed to be 18 to 60 [29]. Because the life expectancy of children with HIV/AIDS is 28.79 years [25], the effective labour years were assumed to be 10.79 years, and the potential labour years lost was estimated to be 31.21 years for a single paediatric HIV case. People living with HIV tend to experience unemployment; according to an international labour organization, the unemployment rate of HIV-infected persons was more than three times the unemployment rate of normal individuals in the country [30]. Based on previous studies [31, 32], we speculated that the unemployment rate of HIV-infected children when they grew to an effective labour force age was more than five times that in the country, which was 20.5% (4.1%*5) [33]. Second, the present value of forgone lifetime earnings (E) was estimated by the human capital approach developed by Max and colleagues [34, 35]. A discount rate (c_*discount*_) was used to convert a stream of earnings into its current worth. To consider the potential growth of future earnings, we assumed an annual productivity growth rate of 8.5%, which is approximately the growth rate of the GDP per capita in Yunnan province in 2016 [36]. Third, we considered weighting the annual earnings using an income distribution coefficient (c_*income*_) due to the differences in the different age groups [37]. The equation for lifetime forgone earnings (E) was as follows:

$$ {E}_{HIV+}=\sum \limits_{Y=18}^{Y=28.79}\left[ GDP\left({Y}_{2015}\right)\times {\left(1+0.085\right)}^{Y-2015}\times {c}_{income}\right]/{\left(1+{c}_{discount}\right)}^{Y-2016} $$

$$ {E}_{\mathrm{normal}}=\sum \limits_{Y=18}^{Y=60}\left[ GDP\left({Y}_{2015}\right)\times {\left(1+0.085\right)}^{Y-2015}\times {c}_{income}\right]/{\left(1+{c}_{discount}\right)}^{Y-2016} $$

We estimated the return-on-investment (ROI) as a ratio, with total savings (numerator) divided by the total investment (denominator) [37].

### Sensitivity analysis

One-way sensitivity analysis was conducted to examine the impact of changes in model assumptions. First, we assessed the impact of changing the HIV MTCT rate in the pre-PMTCT era to its largest and smallest values (20%~ 45%) reported in the previous literature [1]. Second, we changed the growth rate of GDP per capita in Yunnan province to the lowest rate in China in the past ten years with 6.4% (2015) and to nearly the highest rate in China in the last ten years with 12.1% (2006) [38]. Third, we assessed the impact of changing life expectancy values to their upper (69.4years) [39] and lower limits (14.23 years) [40]. Fourth, we set the discount rate for converting investment and economic costs to its largest and smallest values at 0.0 and 5.0%, respectively. Finally, we performed a two-way sensitivity analysis. All parameter values used in the analysis are presented in Table 2.

**Table 2:** Parameters and ranges for analysis

Variable	Base	low	high	source
Mother-to-Child transmission rate (%)				
Pre-PMTCT era	34.80	20.00	45.00	surveillance data; [1]
Pro-PMTCT era				
2006	8.78	–	–	surveillance data
2007	11.36	–	–	surveillance data
2008	6.73	–	–	surveillance data
2009	8.62	–	–	surveillance data
2010	6.49	–	–	surveillance data
2011	4.47	–	–	surveillance data
2012	4.36	–	–	surveillance data
2013	4.19	–	–	surveillance data
2014	3.16	–	–	surveillance data
2015	3.13	–	–	surveillance data
Total number of HIV-infected pregnant women in the particular years				
2006	923			surveillance data
2007	1043			surveillance data
2008	1236			surveillance data
2009	1702			surveillance data
2010	1723			surveillance data
2011	1867			surveillance data
2012	1940			surveillance data
2013	1904			surveillance data
2014	2045			surveillance data
2015	2011			surveillance data
2006–2015	16,394			surveillance data
Health Utility				
General population	1.00	–	–	
Patient with HIV	0.74	–	–	[24]
Average life expantancy (Yunnan Province, 2015)	70.50	–	–	[26]
Cost(2016,US$)				[27]
ART per person per year	2947.94	–	–	
Regular follow-up and testing per person per year	94.46	–	–	
Uptoward effect treatment per person per year	287.74	–	–	
Opportunistic infection per person per year	231.30	–	–	
Total direct cost per year per person	3561.44	–	–	
Coefficient for direct cost of infant:adult	1.75:1			
Total direct cost per year per infant	6232.51	–	–	
Life expentancy of newborn of HIV with treatment	28.79	14.23	69.40	[25, 39, 40]
Coefficient for unemployment rate of HIV positive:general	5:1			[30–32]
Average unemployment rate in China	4.10%			[33]
GDP per capita in 2015 ($US)	4222.66	–	–	[36]
Annual rate of growth of GDP per capita	8.50%	6.50%	12.10%	[36, 38]
Discount rate	3.00%	0.00%	5.00%	[22, 23]

## Results

### Investment in PMTCT

The total investment over the 10-year study period as well as investment stratified by calendar year and investment sources (e.g., Chinese Central Government, International Aid) are presented in Table 3. The total investment increased from US$3.9 million in 2006 to US$5.1 million in 2015 (a 38.5% increase over 10 years).

**Table 3:** The investments per year from 2006 to 2015 (US$, 2016)

year	Chinese Central Government	Local Administration	International Aid	Social Finacing	Total Investment
2006	3,674,879	162,323	94,266	0	3,931,468
2007	3,599,579	260,445	124,372	4353	3,988,749
2008	2,811,680	294,274	140,639	4353	3,250,946
2009	2,979,986	417,998	132,723	726,258	4,256,965
2010	4,928,218	470,442	77,174	653,137	6,128,971
2011	5,561,483	288,188	6843	0	5,856,514
2012	5,514,498	189,945	6419	0	5,710,862
2013	4,879,138	197,393	5242	10,065	5,091,838
2014	5,293,116	187,487	0	18,730	5,499,333
2015	5,085,509	218,356	0	155,129	5,458,994
2006–2015	44,328,086	2,686,851	587,678	1,572,025	49,174,640

### Savings generated by PMTCT

Table 4 presents the estimated number of paediatric HIV infections averted by the PMTCT programmes in Yunnan Province from 2006 to 2015 as well as the investment required per infection averted. Overall, we estimated that approximately 2725 new infections were averted at an estimated US$18,045 investment required per infection averted. Similarly, Table 4 also shows the QALY acquired and investment required per QALY acquired. Overall, a total of 134,078 QALY were acquired at an investment per QALY of US$367.

**Table 4:** Savings of PMTCT (US$, 2016)

year	Number of pediatric HIV infections averted	Investment per pediatric HIV infections averted	Total QALYs acquired	Investment per QALY acquired
2006	144	27,387	7063	557
2007	148	26,883	7301	546
2008	209	15,588	10,261	317
2009	246	17,335	10,282	352
2010	280	21,890	13,775	445
2011	312	18,765	15,355	381
2012	325	17,550	16,010	357
2013	336	15,136	16,551	308
2014	367	14,984	18,056	305
2015	358	15,241	17,623	310
2006–2015	2725	18,045	134,078	367

Table 5 shows the direct, indirect, and total economic costs saved by PMTCT based on the estimated number of paediatric HIV infections averted. In total, we estimated that Yunnan’s PMTCT programmes saved US$0.5 billion to US$4.4 billion in direct costs (i.e., treatment provided) and US$3.9billion in indirect costs (i.e., lost productivity). On average, we estimated that for every paediatric HIV infection averted, the healthcare system saved US$179,444 in treatment costs and society gained US$1,433,310 in productivity.

### Return on investment

As shown in Table 5, we found that the ROI ratio was more than 45 every year during the study period, meaning that for every US$1 invested in PMTCT in Yunnan province, more than US$45 was saved by avoiding spending on treatment costs and loss of productivity. The total ROI ratio was 88.4 during the study period, meaning that for every US$1 invested in PMTCT in Yunnan province during the study period, more than US$88.4 were saved by avoiding spending on treatment costs and loss of productivity.

**Table 5:** Savings of PMTCT (US$, 2016)

Year	Direct costs saved	Indirect costs saved	Total costs saved	ROI of direct benefit	ROI of indirect benefit	ROI of total benefit
2006	25,757,745	154,660,310	180,418,055	5.6	38.3	44.9
2007	26,623,550	168,395,173	195,018,723	5.7	41.2	47.9
2008	37,422,765	249,340,014	286,762,779	10.5	75.7	87.2
2009	44,063,311	309,261,465	353,324,777	9.4	71.7	82.0
2010	50,238,980	371,434,400	421,673,380	7.2	59.6	67.8
2011	56,000,568	436,140,570	492,141,138	8.6	73.5	83.0
2012	58,388,457	479,019,957	537,408,415	9.2	82.9	93.1
2013	60,362,285	521,655,858	582,018,142	10.9	101.5	113.3
2014	65,856,571	599,527,566	665,384,137	11.0	108.0	120.0
2015	64,271,038	616,335,350	680,606,389	10.8	111.9	123.7
2006–2015	488,985,271	3,905,770,664	4,394,755,935	8.9	78.4	88.4

### Sensitivity analysis

**Fig. 1:**
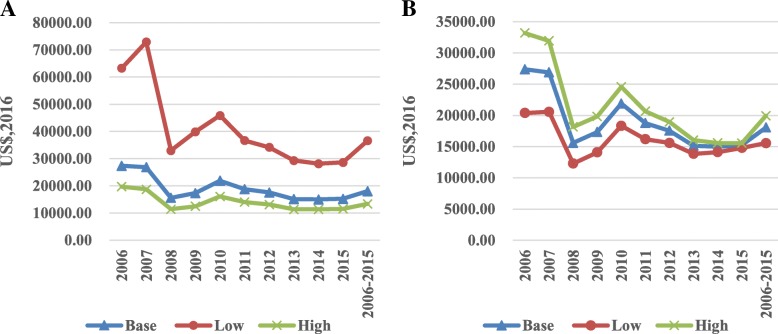
**a** the sensitivity analysis of investment per pediatric HIV infection averted by changing HIV MTCT rate in the pre-PMTCT period. **b** the sensitivity analysis of investment per pediatric HIV infection averted by changing discount rate

**Fig. 2:**
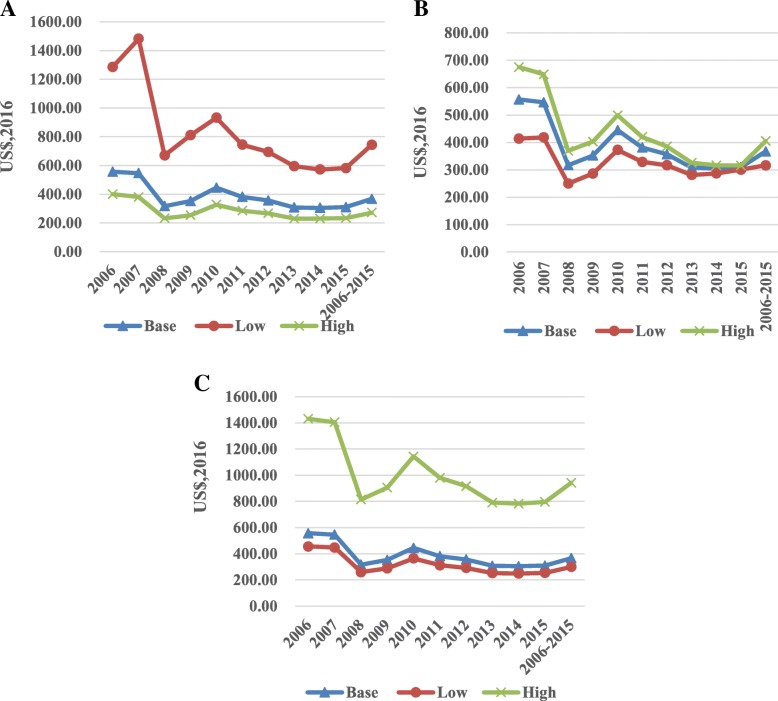
**a** the sensitivity analysis of investment per investment per QALY acquired by changing HIV MTCT rate in the pre-PMTCT period. **b** the sensitivity analysis of investment per investment per QALY acquired by changing discount rate. **c** the sensitivity analysis of investment per investment per QALY acquired by changing life expectancy.

**Fig. 3:**
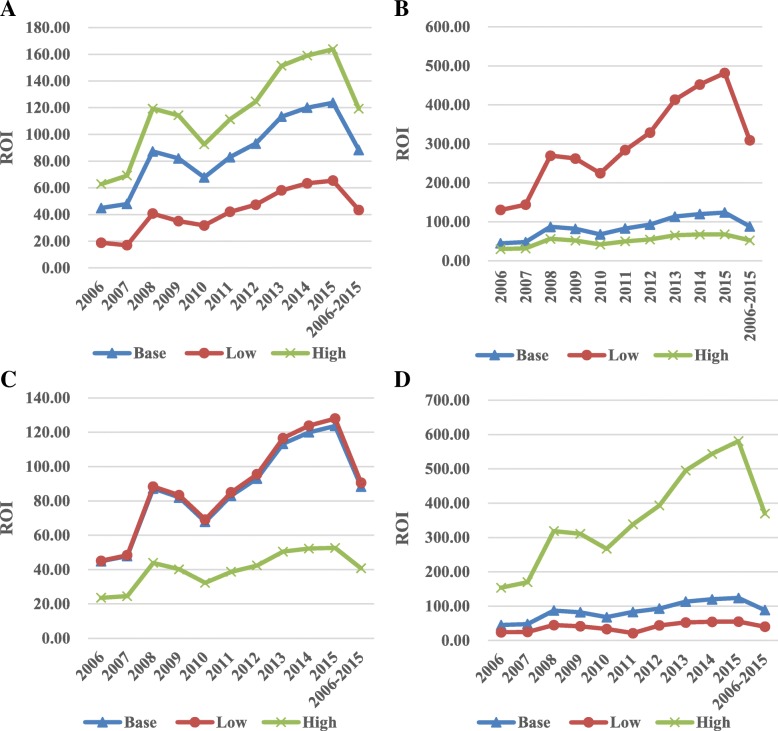
**a** the sensitivity analysis of ROI by changing HIV MTCT rate in the pre-PMTCT period. **b** the sensitivity analysis of ROI by changing discount rate. **c** the sensitivity analysis of ROI by changing life expectancy. **d** the sensitivity analysis of ROI by changing growth rate of GDP in Yunnan province

When we increased the HIV MTCT rate during the pre-PMTCT period from 34.8 to 45.0%, the investment per paediatric HIV infection averted declined from US$18,045 to US$13,370. The investment per QALY acquired also declined from US$367 to US$272. In contrast, the ROI ratio increased from 88.4 to 119.3. When we increased the discount rate from 3.0 to 5.0%, the investment per paediatric HIV infection averted increased from US$18,045 to US$19,935, the investment per QALY acquired increased from US$367 to US$405, and the ROI ratio declined from 88.4 to 52.5. When we increased the life expectancy from 28.79 years to 69.4 years, the investment per QALY acquired increased from US$367 to US$943, and the ROI ratio decreased from 88.4 to 40.7. Lastly, by changing the GDP growth rate in Yunnan province from 8.5 to 12.1%, making it the second highest growth rate in China in the last ten years, the ROI ratio increased from 88.4 to 369.9 (Figs. 1, 2, 3). As shown in Table 6, when we changed the two assumptions simultaneously, the investment required per paediatric HIV infection averted varied from US$11,530 to US$40,461, the investment per QALY acquired varied from US$223 to US$1913, and the ROI ratio varied from 19.3 to 1708.6.

**Table 6:** The muiti-way sensitivity analysis (US$, 2016)

	Investment per pediatric HIV infections averted	Investment per QALYs acquired	ROI
Min value	11,530	223	19.3
Max value	40,461	1913	1708.6

## Discussion

We found that the US$49.17 million investment in Yunnan’s PMTCT programmes over the period from 2006 to 2015 averted an estimated 2725 new paediatric HIV infections and resulted in an estimated 134,008 QALY acquired. It furthermore saved an estimated US$0.5 billion in treatment expenditures for Yunnan’s healthcare system and nearly US$4 billion in productivity. On a per case basis, we estimated that for every US$18,005 invested, one paediatric HIV infection could be avoided, which meant that an estimated US$0.2 million in healthcare costs and US$1.4 million in lost productivity would be saved. On a per QALY basis, we estimated that for every US$367 invested, one QALY was gained.

Considering the most significant route of HIV infection among children in developing countries [41] and the rapid transmission of HIV between the two generations is MTCT, it was therefore urgent to implement PMTCT programmes for pregnant women living with HIV and their newborns. Based on the economic and development situations, putting available PMTCT resources to optimal use is essential. From 2006 to 2015 in Yunnan province, nearly US$50 million was invested in PMTCT programmes. After 2010, an annual investment of more than US$5 million averted more than 300 paediatrics HIV infections per year. Given the investment per paediatric HIV infection averted and the investment per QALY acquired in 2013, 2014 and 2015, this is considerably lower than was made available for PMTCT in previous years, suggesting that PMTCT programmes were more cost-effective in this period. In this period, the ART regimens changed to a triple therapy. The national-provincial-city level-county-level network has maturely developed. The capacity of the organizations to implement, manage and monitor the PMTCT programmes has greatly advanced. This finding is consistent with an empirical evaluation of PMTCT programmes in Zambia in 2014, which also demonstrated the cost-effectiveness of PMTCT programmes [42].

Our study also suggested that Yunnan’s PMTCT programmes saved US$4.4 billion - US$0.5 billion in direct costs and US$3.9 billion in indirect costs in ten years based on the cost-benefit analysis. From a financial perspective, this study demonstrated that the investment in PMTCT programmes made good financial sense. The ROI was 88.4, meaning that every US$1 invested brought about US$88.4 in benefits, which was more than some previous studies’ results about ROI, such as 1.0~40.0 in the other HIV/AIDS programmes [43–45]. In other words, the generated benefits of the investment in PMTCT programmes were greater than the investments, and we could consider the investments in PMTCT programmes to be cost-efficient. In particular, the cost-efficiency was more noticeable in recent years (more than US$0.6 billion was saved).

Given the robustness of our cost-effective measures after examining their sensitivity after changing a variety of parameters, including the HIV MTCT rate in the pre-PMTCT period, discount rate, life expectancy of HIV-infected infants and the GDP growth rate of Yunnan province, we consider this cost efficiency of the PMTCT programmes to be credible. We also found that the investment per QALY acquired increased with the increased life expectancy of people living with HIV due to lifelong ART. This means that PMTCT programmes in the ART period could require more investment. Because of the rapid growth of economics in China and based on economic development in Yunnan province, we supposed a high GDP growth rate of more than 10%, which approaches the highest growth rate in China in the last 10 years [38]. In this case, the benefits of the investment in PMTCT programmes increased greatly. If we successfully avert one newborn from being infected with HIV, a healthy child will yield more productivity to society when he or she grows to the age with effective labour force under this development situation. The optimization results supported the ongoing investments in effective PMTCT programmes in the future.

Cost-effectiveness and cost-benefit analysis above could provide meaningful information about the economic value of PMTCT strategies and enable optimal allocative efficiency analyses to highlight the gains that have been achieved by targeting the constrained resources to the right interventions. The investment in PMTCT programmes would not only generate benefits for the children but also for the mothers, which could help detect undiagnosed infections and allow mothers living with HIV to receive life-saving ART immediately. If this benefit was to be included, the final results would be more positive.

From 2006 to 2015, PMTCT programmes have been scaled up and extended in Yunnan province, and the rate of mother-to-child transmission has decreased substantially. Currently, as the first country in the world to declare “triple elimination certifications”, China is on the road to eliminate the transmission of HIV, hepatitis B and syphilis from mother-to-child. Importantly, Yunnan province began to initiate this work. We aim to eliminate the transmission of HIV from mother-to-child during the 13th five-year plan (from 2016 to 2020) [46]. Therefore, investing in PMTCT programmes and advancing the use of investments will build a brighter future for children, mothers, provinces and the nation.

Our study had several limitations. First, we conducted an economic evaluation on the macro-level and an ecological study using aggregate-level data. The ecological evaluation did not identify the heterogeneity among individuals and could not control the confounding factors. Second, we used the investment as the cost to do the analysis; although all the expenditures on the PMTCT programmes were earmarked funding for their specific purposes alone, it could over-estimate the net cost. Third, no study has reported the life expectancy of HIV-infected children in China. We used the life expectancy of HIV-infected children in Africa reported in the published study to substitute for this finding. Some differences may exist among different countries regarding the life expectancy of HIV-infected children. Fourth, our study did not consider the influence of other HIV/AIDS prevention programmes. Finally, we used QALY acquired in the investment-utility analysis, but using QALY to estimate the utility is sometimes subjective. Finally, the loss of productivity may be overestimated based on the PMTCT programmes because some other HIV prevention programmes may exist over the lifetime. Despite these limitations, we believe that our study thoroughly quantified the historical economic value of the PMTCT programmes carried out in Yunnan province based on the perspective of a policymaker and showed the cost-effective and cost-benefit of the PMTCT programmes from 2006 to 2015. We believe that our study used the scientific method to verify the PMTCT programmes’ provisions from an economic point of view to help make the resource allocation strategy more evidence-based.

## Conclusion

Our results support the ongoing investment in PMTCT programmes in Yunnan Province. PMTCT strategies were a cost-effective and cost-benefit strategy from 2006 to 2015. Despite requiring higher investments in the future, the overall investment in PMTCT programmes in Yunnan province could be offset by averting more paediatric infections. When we achieve the goal of eliminating the transmission of HIV from mother-to-child during the 13th five-year plan, investing in PMTCT programmes and advancing the efficiency of the investments using will build a brighter future for children, mothers, provinces and the nation.

## Abbreviations

CHEERS: Consolidated health economic evaluation reporting standards
MTCT: Mother-to-child transmission
PMTCT: Prevention of mother-to-child transmission
QALY: Quality of life years
ROI: Return-on-investment ratio
WHO-CHOICE: World Health Organization CHOosing Interventions that are Cost-Effective
